# Patient dissatisfaction following rhinoplasty: a 10-year experience in Iran

**DOI:** 10.1186/s40902-022-00369-z

**Published:** 2023-01-26

**Authors:** Farhad Ghorbani, Hanie Ahmadi, Ghazal Davar

**Affiliations:** 1 Department of Oral and Maxillofacial Surgery, Shiraz University of Medical Sciences, Shiraz, Iran; 2 Student Research Committee, Shiraz University of Medical Sciences, Shiraz, Iran

**Keywords:** Nose, Rhinoplasty, Dissatisfaction, Complaints, Forensic medicine

## Abstract

**Background:**

The nose is one of the most prominent parts of the face and plays a significant role in peoples’ self-satisfaction as well as quality of life. Rhinoplasty is considered as one of the most numerous and delicate cosmetic surgeries all around the world that can be performed for functional issues, esthetic issues, or both. In this study, we aimed to evaluate the dissatisfaction of patients who had undergone rhinoplasty surgery and inform the surgeons to improve the surgical techniques to prevent probable future complaints.

**Method:**

This retrospective cross-sectional study was conducted to report various aspects of dissatisfaction of patients following rhinoplasty. All available files in the Fars Forensic Medicine Department between 2011 and 2020 were reviewed, and the required information was extracted.

**Results:**

Out of 117 patients, 68.4% were females and 31.6% were males. Most of the patients were in the age range of 30–34 years. In terms of educational attainment, the highest frequency is associated with academically educated patients and the lowest with a diploma. The majority of cases filed for litigation less than 6 months after their rhinoplasty. The first rank among the factors of dissatisfaction with surgeries belonged to “respiratory problems” (36.8%) followed by “dissatisfaction with the general shape of the nose” (34.2%).

**Conclusion:**

Our study shows that middle-aged female patients may be more difficult to satisfy. In general, at younger ages, patients complain about esthetics, and with aging, most patients feel dissatisfied with nasal function.

## Background

Nose is the major portal of the respiratory and one of the most important organs in the human body [1]. The nasal complex is divided into two parts regarding function and location [2]. The external nose is a pyramidal structure, situated in the midface, with its base on the facial skeleton and its apex projecting anteriorly, while two symmetrical bones at the top and two sets of paired cartilage at the bottom support this structure [2]. On the inside, the human nose is composed of several layers of ciliated epithelial cells covered with a mucous blanket throughout the nasal cavity [3].

Nasal complex and related structures play an important role in the functions that include purifying, warming, and humidifying the inhaled air as well as directing exhaled air out and providing local immunity [2, 4]. In addition, a desired nasal feature can improve the individuals’ esthetic and self-confidence additionally [5]. To improve nasal appearance and function, multidimensional and extensive surgeries have been designed including different types of rhinoplasties [6].

Rhinoplasty is considered one of the most numerous and delicate cosmetic surgeries all around the world [7]. These surgeries involve alteration in the bony and cartilage structures of the nose and can result in the elimination of nasal deviation and asymmetries, elimination of respiratory problems, removal of nasal bridge humps, and shaping of alas [8]. Surgical access to the nose can be gained via close rhinoplasty, open rhinoplasty, or a combination of the two [9]. The goal of rhinoplasty is to improve the nose esthetically as well as functionally [6].

Furthermore, patients’ dissatisfaction has been observed in rhinoplasty as well as other cosmetic surgeries [5]. Not paying attention to patients’ chief complaints can lead to a rise in the number of dissatisfied patients [10]. Patients can be dissatisfied with the overall shape of their nose, airway obstruction and breathing quality, nasal nostril forms, and scar formation after their nose surgeries [10].

There are numerous ways to measure the function and esthetic outcome after rhinoplasty, for example, anatomical and physiological factors, the patient’s reported nasal symptoms, psychological function and satisfaction with health care, quality of life after the surgery, and rhinoplasty revision rates [11, 12].

Patients’ complaints are one of the most serious and stressful job-related problems that any physician faces during his/her career life. Nowadays, regarding the increase in rhinoplasty popularity, patients’ complaints have been reported more than previously [13]. Also, few accurate studies have been done about the causes and consequences of the patients’ dissatisfaction following surgeries. In this study, we aimed to evaluate the dissatisfaction of patients who had undergone rhinoplasty surgery and inform the surgeons to improve the surgical techniques to prevent probable future complaints.

### Method

This retrospective cross-sectional study was conducted to report various aspects of dissatisfaction of patients following rhinoplasty. The study protocol was approved by the Medical Ethics Committee of Shiraz University of Medical Sciences with an ethical number of IR.SUMS.DENTAL.REC.1399.222. Human dignity, medical confidentiality, and adherence to Helsinki ethical rules have been considered throughout the study.

All files containing rhinoplasty surgeries complaints were collected from Fars province Forensic Medicine department archive between January 2011 and December 2020. The required information including the patients’ demographic data, the time elapsed from surgery to complaint registration, and types of dissatisfaction was extracted by reviewing the all-archived files; therefore, there was no need for sampling.

All of the complete complaint files about rhinoplasty that were archived from January 2011 to December 2020 were included in this study. Incomplete files and files related to patients with systematic disorders, facial defects, previous trauma, and congenital syndromes which can play a role in increasing postoperative complications were excluded from the study. The principle of information confidentiality was observed throughout the study, and written informed consent was obtained.

A trained person who was unaware of the information of the participants gathered the data. Microsoft Excel sheets have been used to create the tables and graphs. Also, chi-square test was used to find any significant association between the parameters, and a *P*-value < 0.05 was considered as statistically significant. The analysis was performed using Statistical Package for the Social Sciences (SPSS) version 24.0 software.

### Results

Overall, 117 cases were evaluated. Out of the 117 patients, 37 (31.6%) were male and 80 (68.4%) were females. After dividing the age range of patients into 5-year periods, it was found out that the lowest complaints were for the 19–24-year-old age range with 18 (15.4%) individuals, the age range of 25–29 years had the highest number with 25 people (21.4%), 29 (24.8%) were in the age range of 30–34, 22 (18.8%) were in the age range of 35–39 years, and 23 (19.7%) were above 40 years. The mean age of those who registered complainants about their rhinoplasty was 32.86 years with a standard deviation of 8.48. Table 1 shows the demographic data of patients.

**Table 1:** The demographic features of the participants (*N* = 117)

Variable	Frequency	Percentage
Sex		
Male	37	31.60%
Female	80	68.40%
Age		
19–24 years	18	15.40%
25–29 years	25	21.40%
30–34 years	29	24.80%
35–39 years	22	18.80%
Above 40	23	19.70%
Marital status		
Married	57	48.70%
Single	60	51.30%
Education level		
High school and less	47	40.20%
Academic education	70	59.80%
Time elapsed between rhinoplasty and complaint registration		
Less than 6 months	36	30.80%
6 months to 1 year	24	20.50%
1 to 2 years	25	21.40%
2 to 3 years	11	9.40%
3 to 4 years	8	6.80%
4 to 5 years	5	4.30%
More than 5 years	8	6.80%
Total	117	100%

More than half of the patients involved in our study were single (*N* = 60, 51/3%), and 57 (4.7%) were married. Although there is no significant difference between the two groups (*P*-value > 0.05). More than half of the patients (*N* = 70, 59.8%) had academic education, and 47 (40.2%) were not graduated from the university. Table 1 shows the patients’ demographic data. In our study, the time intervals between rhinoplasty and litigation in cases related to rhinoplasty complaints were divided into 7 categories: less than 6 months, 6 months to 1 year, 1 to 2 years, 2 to 3 years, 3 to 4 years, 4 to 5 years, and above 5 years. The minimum amount of time elapsed between rhinoplasty and the complaint registration was 2 months after surgery, and the maximum time interval in this study was 12 years. The highest complaints were reported less than 6 months after surgery (*N* = 36, 30.8%), and the lowest complaints stated 4 to 5 years after rhinoplasty (*N* = 5, 4.3%) (Table 1).

We also divided the causes of the patients’ dissatisfaction into two groups of functional and esthetic problems. Nasal functional problems include breathing difficulties, olfactory problems, and rhinitis. Among functional problems, the greatest number of complaints was related to breathing difficulties (*N* = 43, 36/8%). Nasal esthetic problems include nasal deviation, dissatisfaction with the nasal overall shape, nostril asymmetry, nasal tip malformation, skin deformity due to scar, ala malformation, and nasal bridge malformation after the surgery. Among esthetic problems, the greatest number of complaints was related to dissatisfaction with nasal overall shape (*N* = 40, 34/2%) followed by nostril asymmetry (*N* = 34, 29/1%). Table 2 shows the distribution of the patients’ complaints.

**Table 2:** Patients’ complaints after rhinoplasty

			Frequency	Percentage
**Complaint’s cause**	**Functional**	Breathing difficulties	43	36.80%
		Olfactory problems	19	16.20%
		Rhinitis	14	12%
	**Esthetic**	Nasal deviation	21	17.90%
		Dissatisfaction with the nasal overall shape	40	34.20%
		Nostril asymmetry	34	29.10%
		Nasal tip malformation	28	23.90%
		Skin deformity due to scar	27	23.10%
		Ala malformation	13	11.10%
		Nasal bridge malformation	15	12.80%

**Fig. 1:**
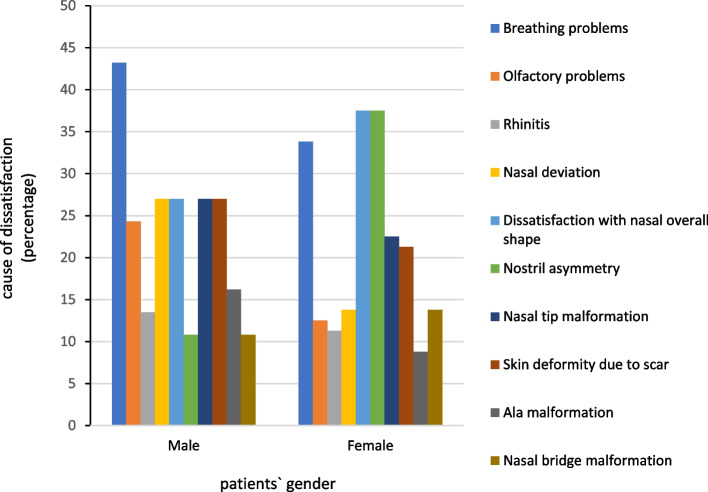
The relationship between the patients’ gender and their cause of dissatisfaction following rhinoplasty

Among male patients, nasal obstruction and breathing problems recorded were the most dissatisfaction complaints (*N* = 27, 43.2%). Dissatisfaction with nasal overall shape and nostril asymmetry had the highest amount of dissatisfaction among female patients (*N* = 30, 37.5%). Figure 1 shows the relationship between the patients’ gender and their reason to sue the surgeon. The relationship between the patients’ gender and their esthetic or functional aspect of complaints was not statistically significant (*P* > 0.05).

**Fig. 2:**
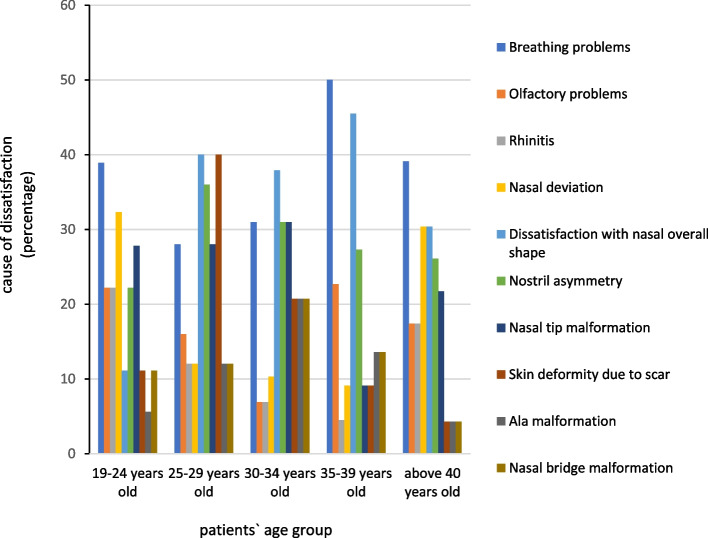
The relationship between the patients’ age group and their cause of dissatisfaction following rhinoplasty

Among 19–24-year-old patients, breathing problems had the highest rate of dissatisfaction (*N* = 7, 38.9%) complaints. Dissatisfaction with nasal overall shape and skin deformity due to the scar had the the highest rate of dissatisfaction among 25–29-year-old patients (*N* = 10, 40%). Among 30–34-year-old patients, the nasal overall shape had the highest rate of dissatisfaction (*N* = 11, 37.9%). Among 35–39-year-old and above 40-year-old patients’ breathing problems had the highest rate of dissatisfaction with 11 (50%) and 9 (39.1%) complaints, respectively. Figure 2 demonstrates the relationship between the patients’ age group and their cause of dissatisfaction following rhinoplasty. The relationship between the patients’ age and their esthetic or functional complaints was not statistically significant (*P* > 0.05).

**Fig. 3:**
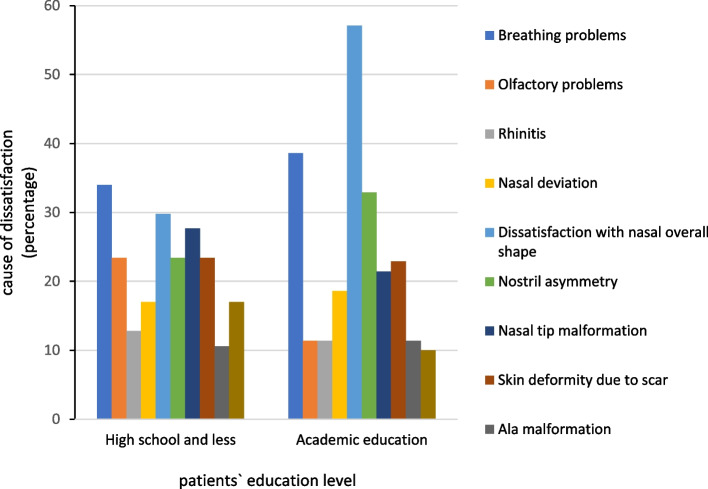
The relationship between the patients’ education level and dissatisfaction after rhinoplasty

Among the patients with no academic education, breathing problems had the highest rate of dissatisfaction (*N* = 16, 34%). Additionally, the nasal overall shape had the highest rate of dissatisfaction among the university-educated patients (*N* = 26, 57.1%) (Fig. 3). The relationship between the patients’ education level and their esthetic or functional complaints was not statistically significant (*P* > 0.05).

**Fig. 4:**
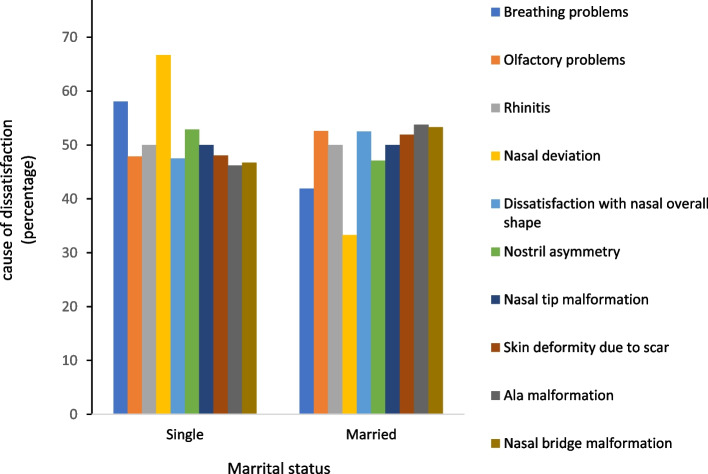
The relationship between the patients’ marital status and dissatisfaction after rhinoplasty

Among single patients, nasal deviation had the highest rate of dissatisfaction (*N* = 14, 66.7%). The reasons for dissatisfaction among married patients revealed a homogenous distribution; nevertheless, dissatisfaction with nasal ala malformation had the highest rate (*N* = 11, 53.8%). Figure 4 shows the relationship between the patients’ marital status and their dissatisfaction. Also, the relationship between the patients’ marital status and their esthetic or functional aspect of complaints was not statistically significant (*P* > 0.05).

**Fig. 5:**
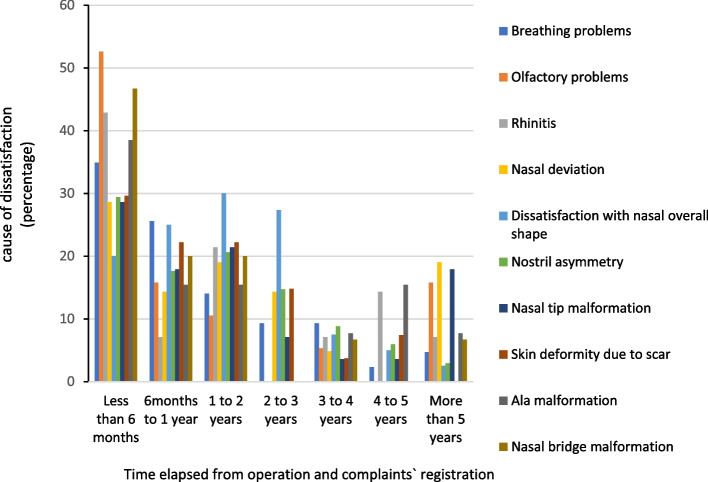
The relationship between the time elapsed from the surgery and complaints’ registration and their dissatisfaction after rhinoplasty

Among patients who registered their complaints in less than 6 months after their rhinoplasty, olfactory problems and nasal bridge malformation were the most reported reasons with 10 (52.6%) and 9 cases (46.7%), respectively. Among patients who registered their complaints 6 months to 1 year after their rhinoplasty, breathing problems were the most reported reasons (*N* = 1, 25.6%). Among the patients who registered their complaints in 1 year to 2 years after their rhinoplasty, nasal overall shape dissatisfaction was the most reported reason (*N* = 12, 30%) to complain. Among those who registered their complaints 2 to 4 years after their rhinoplasty, dissatisfaction reasons almost illustrated a homogenous distribution. Among complaints in 4 to 5 years and more than 5 years after the rhinoplasty, nasal ala malformation and nasal deviation dissatisfaction were the most reported reasons with 2 (15.4%) and 4 (19%) complaints, respectively. Figure 5 shows the relationship between the time elapsed from operation and registration of the complaints. The relationship between the time elapsed from the operation and registration of the complaints and their esthetic or functional aspect of complaints was not statistically significant (*P* > 0.05).

### Discussion

The nose plays a significant role in people’s quality of life due to its prominent position in the face and its high importance in terms of providing respiratory function, esthetics, and olfactory ability [9]. The need for changes in the nasal function and esthetics is more important than ever considering the increasing rate of people’s communication with each other in today’s life, widespread use of social networks, high importance of perfect appearance among people, and the need to increase respiratory efficiency due to air pollution [5].

Nowadays, rhinoplasty accounts for the largest number of head and neck surgeries, whether due to the correction of functional problems of the nose or improvement of its beauty [14, 15]. In 2013, 1,668,420 esthetic surgeries were performed in the US, and some unofficial statistics indicate that in Iran, the rate of esthetic rhinoplasty surgery is seven times that of the USA [14]. Given the sensitivity, importance, and the high number of this elective surgery, it is obvious that the statistics of dissatisfaction and complaints about the results are not negligible [16]. It is clear that complaints against physicians are not true in all cases, but they can have serious and long-term destructive personal and social effects for them and the people around [17].

Ong et al.’s investigation about the effect of patient complaints on surgeons shows that patient complaints may affect the physicians’ occupational status [18]. Physicians try to shorten the duration of their visits, and their desire for early retirement is increasing [19]. Physicians are also less inclined to perform high-risk surgeries, and the number of requests for unnecessary counseling and side examinations is growing [19].

Currently, despite the efforts of the medical staff and the optimal use of available facilities, the number of complaints registered by patients shows an increasing trend, which can be because of population growth, people’s increasing awareness of their rights, increasing insured population, and doctors’ lack of skill to communicate with their patients to meet their needs [19]. Therefore, documents and research that inform the characteristics and sensitivities of patients dissatisfied with the results of their surgery can be very helpful to prevent the consequences of these procedures considering the large number of these complaints [19, 20].

A total number of 117 cases were enrolled in the study, and after categorizing and counting the causes of complaints, the most common concerns expressed by the patients were respiratory problems followed by dissatisfaction with the nasal general shape. Gerhard Rettinger et al.’s study reviewed the risks and complications in rhinoplasty. His studies indicated that 10% of the patients after primary rhinoplasty complained about residual or new breathing problems which were the highest rate among postoperative patients’ dissatisfactions [21, 22]. Among the causes of complaints, nasal ala deformity was the least concern in patients after rhinoplasty. In Gerhard Rettinger et al.’s study, 1–2% of the patients reported postoperative scar problems at the columella, which was the lowest rate among postoperative patients’ dissatisfactions [22, 23].

There is a large difference between the number of men involved in this study who were 37 (31.6%) and women who were 80 (68.4%). These results may be attributed to the fact that women are more sensitive than men about their appearance. In a study on complaints from cosmetic facial surgeries referred to a forensic medicine department in Fars province between 2006 to 2013 conducted by Dr. Kaboudkhani, 55 cases were reviewed, of which 34.5% were males and and 65.5% of the complainants related to women [19].

The youngest person involved in our study was 19 years old, and the maximum age recorded in the files reviewed in this study was 56 years. Also, the majority of people dissatisfied with their rhinoplasty operations were in the age range of 30 to 34 years old. In Kaboudkhani et al.’s study, the youngest person was 20 years old, and the maximum age was 62 years [19]. These results may indicate a wider age range for demanding rhinoplasty surgery in today’s community [24].

Most of the individuals dissatisfied with their rhinoplasty had a university degree. These results are not in the same line with those of previous studies; in the study conducted by Dr. MirAkbari in 2001, about 60% of people had a high school education [25]. Our investigation indicates a higher education level among people undergoing rhinoplasty in recent years and a relationship between higher education and the level of people’s dissatisfaction with their appearance.

Most of the subjects in our study were married, but there was no significant difference between them. This statistic is inconsistent with the results reported in Moradi et al.’s study. They found that 42.6% of cases were married, and 57.4% were single. Overall, in most previous studies, such as ours, the number of single people was higher than that of married people [25, 26].

In our study, the time elapsed between performing rhinoplasty and registration of complaints was also investigated, which is a relatively new approach in this field, and it had not been considered in previous studies. Most of the cases reported dissatisfaction less than 6 months after their operation. The lowest number of complaints is related to the categories of 4 to 5 years and above 5 years. This result may indicate that they are more likely to complain earlier on because that is when they are first seeing the results. Most people would not wait years to make a complaint about their surgery, even though the appearance of the nose is still affected by initial complications such as postoperative swelling and has not yet been fully recovered [25, 27].

The most common cause of complaints among men was the respiratory problem, and dissatisfaction with the general shape of the nose and asymmetry of the nostrils were jointly ranked first among the causes of complaints among women. An Iranian study that evaluated the patients’ chief complaint before rhinoplasty and satisfaction a year after the surgery [28] found that tip drooping was the common complaint among Iranians, and most of the patients wanted to have a natural appearance after rhinoplasty [28]. On the other hand, in another study, facial symmetry improvement was considered as a more challenging limitation to recover esthetics after rhinoplasty [29]. Some studies reported that achieving the ideal postoperative results in the crooked noses is still demanding [30–32].

By reviewing the relationship between the patients’ age and their reason to register complaints, it was found that the most important issues for the 19–24-year-old age group, which is also the youngest group, were respiratory problems and deviation of the nose septum. In the age group of 25 to 29 years, dissatisfaction with the general shape of the nose and the presence of scars had the highest importance among patients. In the above 40-year-old age group, the majority of patients were dissatisfied with respiratory problems. One study in Brazil illustrated that postoperative dissatisfaction was more common among young patients than older ones, and they were more concerned about the outcome of the surgery [29]. For reducing the complications after rhinoplasty, comprehensive information and complete limitation about the procedure should be explained to the patients [29].

Patients with higher education level were mostly dissatisfied with postoperative general form of their nose. On the other hand, high school-graduated and low-educated patients were mostly dissatisfied with postoperative respiratory problems.

In less than the first 6 months after surgery, the predominant causes of dissatisfaction were olfactory weakness and nasal bridge deformity; both can be due to severe swelling of the nose during the first months after surgery and the presence of a nasal dressing, which makes the shape of the patient’s nose unclear and reduces the individual’s olfactory level. Among those who reported dissatisfaction 5 years or more after their rhinoplasty, the most common complaint was nose tip deformity.

Due to the increasing rate of rhinoplasty dissatisfaction, more studies are necessary in the future. The major technical drawback of our study is that we have not examined any CT scan images or nose photography of our cases since we could not access them; therefore, we could not determine if there was a focused selectivity in the reported dissatisfactions. Additionally, an increase in the number of participants of both genders, different age ranges, and other racial population are recommended for further studies. However, we believe that our study represents some important key point for further studies in this field.

### Conclusions

Rhinoplasty remains a complex operation due to the myriad of physical and psychological variables involved. In conclusion, most frequent dissatisfaction in patients receiving rhinoplasty was postoperative respiratory problem followed by unsatisfactory nasal shape. There was no association between dissatisfaction after rhinoplasty and patient age/gender. Most dissatisfied patients have academic status. This can show a direct relationship between the increase in patients’ demand with increasing level of education. However, there was no association between dissatisfaction after rhinoplasty and patient level of education.

Functional problems of the nose after surgery are more important for men, while women are more sensitive to their appearance. In general, it seems that with aging, most patients feel dissatisfied with nasal function after undergoing rhinoplasty.

## Data Availability

The datasets generated during and/or analyzed during the current study are available from the corresponding author on reasonable request.
